# Antibiofilm Efficacy of Calcium Silicate-Based Endodontic Sealers

**DOI:** 10.3390/ma17163937

**Published:** 2024-08-08

**Authors:** Matilde Ruiz-Linares, Vsevolod Fedoseev, Carmen Solana, Cecilia Muñoz-Sandoval, Carmen María Ferrer-Luque

**Affiliations:** 1 Department of Stomatology, University of Granada, 18071 Granada, Spain; drfedoseev@correo.ugr.es (V.F.);; 2 Instituto de Investigación Biosanitaria, 18012 Granada, Spain; 3 Cariology Unit, Department of oral Rehabilitation, Faculty of Dentistry, University of Talca, Talca 3344158, Chile; cemunoz@efom.cl

**Keywords:** AH Plus Jet, AH Plus Bioceramic, biofilms, BioRoot RCS, long-term antimicrobial activity

## Abstract

Background: Using endodontic sealers with long-term antimicrobial properties can increase the success of endodontic treatment. This study aimed to assess the antimicrobial activity over time of two calcium silicate (CS)-based sealers, AH Plus Bioceramic and BioRoot RCS, and to compare them with an epoxy resin-based sealer, AH Plus Jet, against mature polymicrobial biofilms grown on human radicular dentin. Methods: The antimicrobial activity of the sealers was tested using a direct contact test after 1 and 6 weeks of contact with the biofilms. Cell viability was determined by the adenosine triphosphate (ATP) method and flow cytometry (FC). The results of the ATP test were analyzed using an ANOVA with Welch’s correction, followed by the Games–Howell test. The number of cells with damaged membranes obtained by FC in each period was compared by means of an ANOVA and Duncan’s test. For the comparison between times, a Student’s *t*-test was used. Results: Globally, after a week of contact, the epoxy resin-based sealer obtained the best results. However, at 6 weeks, the two CSs showed the highest antimicrobial efficacy, with a significant increase in this activity over time. Conclusions: Calcium silicate-based sealers exert long-term antimicrobial activity against endodontic biofilms.

## 1. Introduction

The aim of endodontic treatment is the prevention and healing of apical periodontitis [1,2]. Using biocompatible filling materials that additionally have antimicrobial properties may be beneficial for endodontic treatment. A bioactive endodontic sealer that hermetically fills the root canal and potentially inhibits the growth of any residual bacteria is desirable [3].

AH Plus^®^ Jet [AH, Dentsply Sirona, Ballaigues, Switzerland] is an epoxy resin-based endodontic sealer that is widely used due to its good physicochemical characteristics, long-term dimensional stability, good adhesion to dentin, fluidity, and biocompatibility [4]. However, its bioactivity and osteogenic potential are limited [5]. Although it has demonstrated some antimicrobial properties, the antiseptic capacity is limited after setting [6]. In addition, mutagenicity, cytotoxicity, inflammatory responses, and hydrophobicity have been reported [7].

Calcium silicate (CS)-based sealers have been gaining popularity, given their bioactivity and biocompatibility [8]. The main components of these materials are calcium silicate, monocalcium phosphate, calcium hydroxide, zirconium oxide, fillers, and thickeners [3,7]. Calcium silicate sealers are hydraulic, and their setting is conditioned by the presence of humidity [9]. Antimicrobial and biomineralization properties are exerted during the setting process through an increasing pH and a release of ions from the material [3,9]. However, these properties can vary greatly depending on the additives in each formulation [8], potentially influencing its indications and clinical application. The first to be marketed were powder–liquid formulations that required manual mixing. More recently, premixed ready-to-use CS formulations have a setting reaction that depends on the moisture existing in the dentinal tubules [9]. Some are still in the early stages of development, requiring more clinical and laboratory studies for their clinical recommendation [7].

BioRoot™ RCS [BR, Septodont, Saint-Maur-des-Fossés, France] is supplied in powder and liquid form. In addition to having good physical properties [10], it has demonstrated low cytotoxicity, inducing the secretion of osteogenic and angiogenic growth factors, and high immunomodulatory properties, which means that it can contribute to the in vivo healing and regeneration process of periapical lesions [11]. It has generally shown antimicrobial capacity in vitro against planktonic bacteria [3] and mono-species biofilms [4,12,13]. Few laboratory studies have evaluated its antimicrobial activity against multispecies biofilms [14,15].

Marketed in 2021, AH Plus^®^ Bioceramic [AHBC, Dentsply Sirona, Ballaigues, Switzerland] is a premixed CS that contains tricalcium silicate, but in a lower percentage than existing ones [16]. The manufacturer claims that it features rapid setting, high wear resistance, and radiopacity; it is safe and biocompatible and does not discolor the tooth. Owing to its recent commercialization, its physical and biological properties are currently being compared with those of other CSs [17,18,19].

To date, one study has evaluated its antimicrobial activity against planktonic cultures of *E. faecalis* [18], with its antibiofilm activity remaining unknown. Therefore, the present experimental study aimed to evaluate and compare the antimicrobial efficacy over time of two endodontic CSs, AHBC and BR, and an epoxy resin-based sealer, AH, against polymicrobial biofilms formed on dentin.

## 2. Materials and Methods

The protocol of this in vitro study was approved by the Ethics Committee of the University of Granada, Spain (N° 1076 CEIH/2020). Informed consent was obtained from all patients prior to the collection of microbiological samples or extracted teeth.

The antimicrobial activity of the sealers over time (1 and 6 weeks) was evaluated using a modified direct contact test (DCT) [20] against polymicrobial biofilms on root dentin (n = 12/group/time). The viability of microorganisms after the DCT was quantified by means of the adenosine triphosphate (ATP) assay and flow cytometry (FC) (n = 10/group/time) [20]. Images obtained by confocal laser scanning microscopy (CLSM) served as an in situ visualization of the residual biofilm in dentin (n = 2/group/time).

The study groups were (1) AH Plus Jet (AH), (2) AH Plus Bioceramic (AHBC), (3) Bioroot RCS (BR), and (4) a positive control (without exposure to any material). Twelve material samples per group and evaluation period (1 and 6 weeks) were prepared. The chemical composition of the sealers and handling instructions, specified by the manufacturer, are summarized in Table 1.

### 2.1. Preparation of Dentin Samples

**Figure 1:**
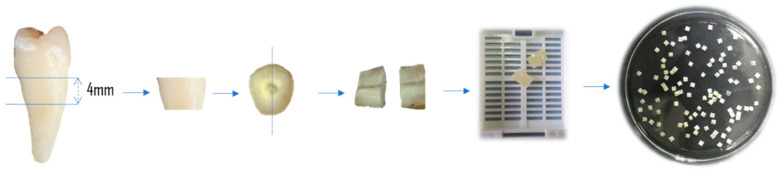
Root dentin sample preparation procedure.

One hundred and twelve sterile specimens of human radicular dentin (4 × 4 × 0.7 mm) from the root coronal third of 56 single-rooted non-carious teeth, extracted for orthodontic reasons, were utilized as substrate for forming biofilms, as previously reported [21]. Briefly, the dentin samples were standardized by cutting with an Accuton-50 machine (Struers, Copenhagen, Denmark), and discarding the middle and apical thirds of the root and the dental crown to obtain a dentin cylinder of the root coronal third. Then, they were sectioned following the root canal lumen, each giving two halves. The root cement was removed by polishing to a flat surface, and the inner face was polished with 150, 220, 500, and 800-grit silicon carbide paper (Figure 1).

To eliminate the smear layer formed during preparation, the samples were immersed in 17% ethylene diamine tetracetic acid (EDTA, DIRECTA AB, Stockholm, Sweden) for 5 min and then washed with saline solution. Subsequently, each of the two halves obtained was randomly assigned to the different study groups. They were then autoclaved and incubated in Trypticase Soy Broth [TSB (Scharlau Chemie SA, Barcelona, Spain)] at 37 °C for 24 h to verify the absence of contamination.

### 2.2. Infection of Dentin Substrates

Microbiological samples were obtained clinically from root canals of necrotic teeth from three volunteer patients following a previous methodology [20] and served as inoculum for dentin infection and biofilm formation. The rubber dam and the tooth were disinfected with 3% H_2_O_2_ and 2.5% NaOCl, which was inactivated with 5% sodium thiosulfate. Pulp chamber access was gained using a sterile round bur, and the chamber was disinfected as previously described. The root canal was filled with sterile saline solution, taking care not to allow it to overflow; a sterile #15 K file (Dentsply Sirona, Ballaigues, Switzerland) was introduced 1 mm short of the apical foramen, and a gentle filing motion was carried out for 30 s before removal. Then, 3 sterile paper points were inserted into the root canal and left inside for 1 min to absorb the fluid. Both files and paper points were placed in microtubes with Tris-EDTA buffer and frozen at −20 °C until use.

For dentin infection, the samples were mixed in TSB and incubated for 24 h at 37 °C in anaerobiosis. Afterward, the cell density was adjusted in a spectrophotometer to a concentration of approximately 3.0 × 10^7^ colony-forming units per milliliter in TSB. Dentin samples were infected in 24-well plates (Corning™, Fisher Scientific, Madrid, Spain) and inoculated with 200 μL of the microbial suspension described above and 1.8 mL of sterile TSB. Sterile dentin blocks were immersed in the inoculated wells and incubated at 37 °C in an anaerobic atmosphere for 3 weeks on a rocking table. The TSB medium was refreshed once a week to ensure the growth of the biofilms. Two dentin samples in each group were analyzed by CSLM to confirm biofilm growth. A negative control group (n = 4) was incubated only with TSB as a sterility control and processed the same way as the other groups.

### 2.3. Antimicrobial Activity Test (DCT)

**Figure 2:**
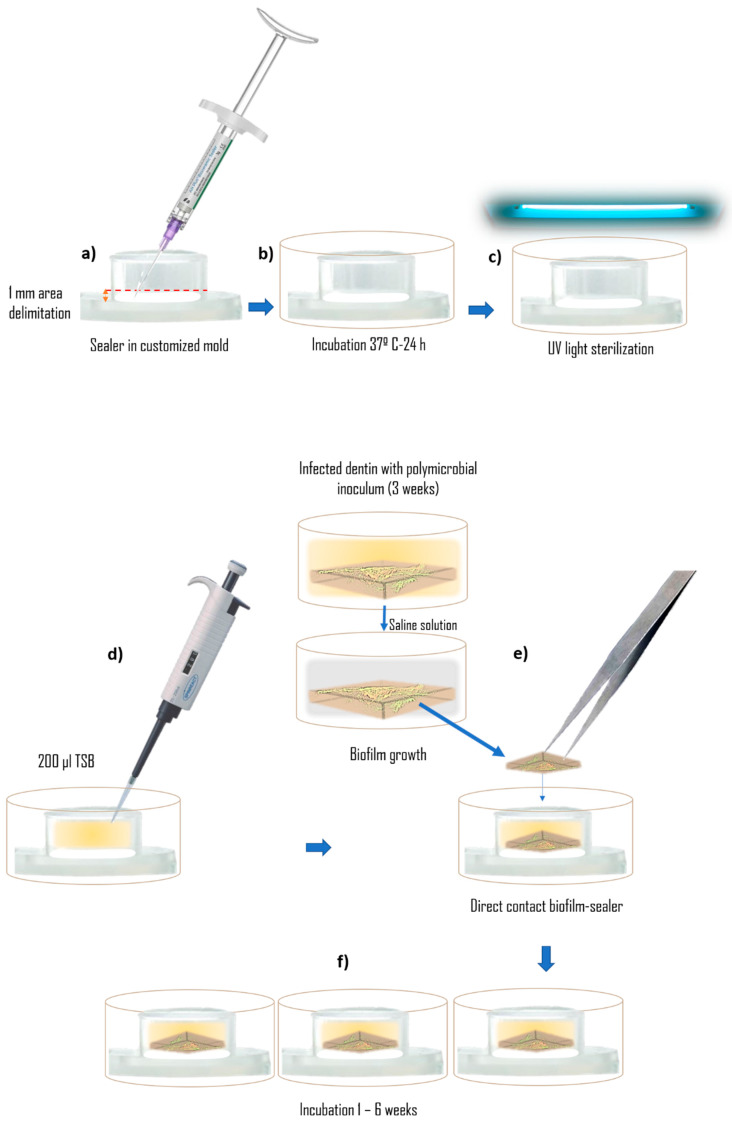
Antimicrobial activity test. Sealers were dispensed in customized mold (**a**), stored for 24 h/37 °C (**b**), and sterilized (**c**). Direct contact test (**d**–**f**).

To evaluate the antimicrobial activity of the materials (Figure 2), against polymicrobial biofilms, the dentin samples with the biofilms formed were put in direct contact with the materials. Under aseptic conditions, equal amounts of each sealer were dispensed in the bottom of the customized molds (1cm Ø × 3mm height). To standardize the volume of sealer in the mold, an area of 1 mm was delimited from its bottom, red dashed line in Figure 2a), and coated with each sealer. Sealers were handled following the manufacturer’s instructions. The materials inside their molds were then introduced into the wells of a 24-well microtiter plate and stored for 24 h in an incubator at 100% humidity for setting. Next, they were sterilized with ultraviolet light, and 200 μL of sterile TSB was added to each mold.

Finally, the dentin blocks with the biofilm formed were placed in direct contact with the materials and incubated for 1 or 6 weeks at 37 °C under anaerobic conditions. Every three days, 100 μL of TSB was added to the molds to prevent desiccation.

After each contact time, 10 dentin blocks (group/time) were separated, placed in microtubes with 200 μL of TSB, stirred for 10 s, and then sonicated on a water-table sonicator (model 5510E-MT; Branson, Danbury, CT, USA) for 10 min to ensure recovery of biofilms. The remaining 2 dentin samples per group were observed under CSLM. For the control group, the same procedure was followed, except that there was no exposure to any material.

### 2.4. Microbial Viability

The cell viability of the recovered bacterial suspensions was evaluated by means of ATP and flow cytometry (FC) [22,23]. The ATP levels contained in the suspension of the recovered biofilms were evaluated with the BacTiter-Glo cell viability assay kit (BacTiter-Glo; Promega, Madison, WI, USA). For this end, 100 μL of the bacterial suspension was added to 100 μL of reagent in a 96-well opaque plate (Greiner, Monroe, NC, USA), followed by incubation at room temperature for 5 min. The luminescence produced was estimated using a luminometer (GloMax™ E6521, Promega, Madison, WI, USA) and expressed as an absolute value of relative light units (RLUs) in each group with respect to the control.

For FC, 100 μL of the microbial suspension was labeled using the LIVE/DEAD cell viability kit (BacLight™; Invitrogen, Eugene, OR, USA) to estimate the integrity of the cytoplasmic membrane. The kit includes two fluorescent nucleic acid dyes with different potentials to penetrate cells. SYTO 9 is a green dye that identifies microorganisms with intact and damaged membranes. Propidium iodide (IP) is a red stain that penetrates only cells with damaged membranes. After staining the microbial suspension with 100 μL of the fluorochromes, the tube was positioned in the FACS Canto II flow cytometer (BD Bioscience, San Jose, CA, USA), and the results were evaluated with the cytometer software (FACSDiva Version 6.1.3., Becton, Dickinson, San Jose, CA, USA). This provided a graph of two-dimensional dots showing the different cell populations within the microbiological sample, which had damaged membranes (considered dead) or undamaged ones (considered viable). Side and forward scatter gates were recognized to exclude debris. In all cases, 30,000 events were evaluated. The results were expressed as an absolute value of damaged/dead cells per milliliter.

For CSLM analysis, dentin specimens were stained with Syto 9/PI (LIVE/DEAD, BacLight; Invitrogen, Eugene, OR, USA) for 15 min [20], rinsed with saline solution, mounted on a 60 l-Dish (Ibidi, Martinsried, Germany) with mounting oil (BacLight™, Invitrogen), and then observed utilizing an inverted confocal laser scanning microscope (Leica TCS-SP5 II, Leica Microsystems, Mannheim, Germany). Microscopic confocal volumes (stacks) from random areas were acquired from each sample using the 40 × oil lens, a 1 μm stepsize, and a format of 512 × 512 pixels.

### 2.5. Statistical Analysis

In previous statistical analyses, the normality of the data was estimated using the Shapiro–Wilk test, and the equality of variances was estimated with the Levene test. When the data followed a normal distribution and the variances were equal, an ANOVA test and a post hoc Duncan’s test were used to show clusters. An ANOVA was performed with Welch’s correction, followed by the Games–Howell test when the variances were not equal. In such cases, a Student’s *t*-test was applied to compare times. Statistical analysis was conducted using SPSS v23.0 (IBM Corp, Armonk, NY, USA). In all instances, a *p*-value < 0.05 was considered significant.

## 3. Results

**Figure 3:**
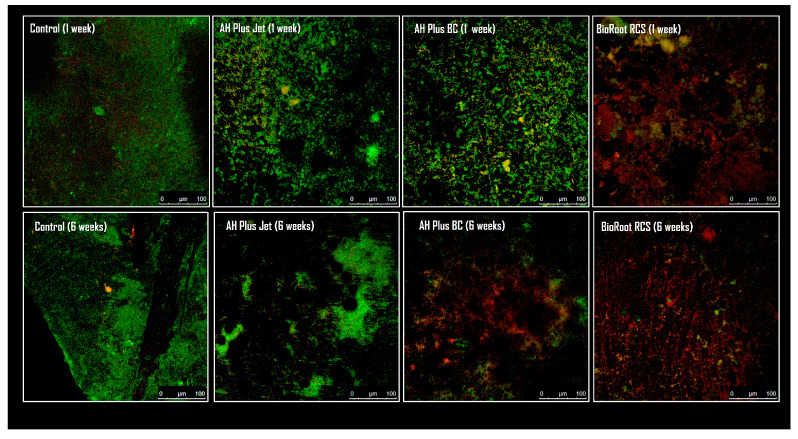
Representative CSLM microphotographs of 3-week biofilms after the direct contact test with the sealers of the different study groups/evaluation time. Syto-9 stained nucleic acid and emitted green fluorescence (considered as live cells), whereas damaged cells were stained by PI (red fluorescence for dead bacteria). The images were consistent with the results obtained in the microbial viability tests.

The results obtained with the ATP test are shown in Table 2. At one week, all sealers except AHBC significantly reduced RLUs with respect to the control (*p* < 0.001). The best results were obtained with AH, followed by BR. After 6 weeks of contact with the biofilms, all sealers obtained a significant decrease in RLUs compared to the control. The behavior of the SC-based sealers evaluated indicated a significant increase in antimicrobial activity over time. The antimicrobial activity of AH increased only slightly from 1 to 6 weeks. Similar findings using FC are shown in Table 3. The microscopic images obtained with CSLM are, moreover, consistent with the results obtained in the feasibility tests (Figure 3).

## 4. Discussion

An ideal endodontic sealer should possess long-term antibacterial ability to reduce the residual bacterial load and prevent or limit microbial growth in the pulp space [3,6]. Because of its recent introduction, the antibiofilm properties of AHBC are still unknown. In turn, BR’s efficacy against polymicrobial biofilms has been poorly evaluated [14,15]. AH was selected as a control.

To the best of our knowledge, this is the first study evaluating the long-term antimicrobial efficacy of AHBC and BR using a clinical endodontic polymicrobial biofilm growth model for 3 weeks [24] and human dentin. A follow-up of antimicrobial efficacy was performed for up to 6 weeks, whereas most research has focused on short-term evaluation [5,13].

To determine antimicrobial activity, a modified direct contact test was employed [20]. This approach is quantitative and reproducible and enables easy standardization; it simulates the contact between the microorganisms of the biofilm growing in the dentin and the applied materials [8]. Microbial viability was evaluated using two reproducible and quantitative approaches that have high sensitivity and specificity [23]. Since ATP is the primary energy molecule in all living cells, its quantification provides an estimate of the viable microbial population in a sample. The CF provides multiparametric information about individual cells within a heterogeneous population, permitting the discrimination of microbes having damaged versus unharmed membranes [23]. In addition, these techniques make it possible to discriminate the population of viable but non-cultivable cells that traditional cultures cannot detect [20]. CLSM also makes available a three-dimensional in situ image of the proportion of viable and non-viable bacteria without disturbing the cells attached to the substrate [24].

In the present study, after 7 days of contact with the biofilms, AH showed lower values of microbial viability according to the ATP test (Table 2), with a decrease in the values of RLUs at 6 weeks. Similar values were obtained with FC, showing only a 3.5% increase in dead cells over time (Table 3). The short-term efficacy of AH may be due to the bactericidal effect of formaldehyde released in small amounts during the setting process or to the toxicity of non-polymerized components [6]. These findings are consistent with those of previous investigations indicating that this sealer does not maintain its antimicrobial activity in the long term [3,6,25]. CSLM images confirmed these findings, with a predominance of cells stained with SYTO 9 in both time periods.

AHBC showed the opposite behavior. This sealer exerted very limited antimicrobial activity at one week, giving the same cell viability as the control group according to both evaluation methods. However, its efficacy over time increased significantly, with a reduction in RLUs of 82% and an increase in cells with damaged membranes (by FC) of around 68% compared to the control at 6 weeks of evaluation. Only one recent research study reports that this sealer could eradicate planktonic E. faecalis within 24 h [18], which are results that do not match those obtained in the present study, given the low activity of AHBC in the short term.

Overall, BR showed the best performance over time. After 7 days, a 37% decrease in RLUs was obtained, which increased to 85% in the last evaluation period, a significant difference between the two evaluated times. Likewise, FC indicated an increase in cells with damaged membranes of around 133% as the observation time increased, with significant differences from the rest of the groups (Table 3). This indicates that BR exerts short-term antimicrobial efficacy that is not lost in the long term. Images obtained by microscopy confirmed these findings. While there was a predominance of cells labeled by SYTO 9 at 7 days and by IP at 6 weeks for AHBC, a greater proportion of cells stained by IP was seen for BR in both time periods evaluated.

The results obtained with CS-based sealers could be related to increased alkalinizing conditions during hydration [13,26]. In the presence of water, calcium silicates form a hydrated calcium silicate gel (CSH, CaO SiO H_2_O), which leads to the formation of calcium hydroxide (Ca(OH)_2_) [8,9]. The dissociation of Ca(OH)_2_ releases calcium (Ca^2+^) and hydroxyl ions (OH^−^), raising the pH. Nonetheless, although an alkaline pH in the microenvironment plays an important role in inhibiting bacterial viability, a significant correlation has been reported between the release of free Ca^2+^ and Si^4+^ ions and the antibacterial effect of SCs [27]. The released ions can cause bacterial membrane depolarization by binding Ca^2+^ and Si^4+^ ions to negatively charged bacterial membrane receptors, resulting in cell lysis [20,27].

The differences observed in this study between the two CSs evaluated over time may be due to their different compositions and the way they are dispensed, directly influencing their mode of hydration, hence the results obtained [8,13]. BR is a material that has a higher amount of CS in its composition than AHBC; and because it is mixed with water, hydration is always guaranteed regardless of the presence of fluids in the environment [13]. The sustained alkalinization of the medium over long periods and its capacity to release high concentrations of Ca^2+^ [4,10,15] leads to lower bacterial viability in the short [12,13] and long term [4], as demonstrated in the present study. On the other hand, it has been suggested that materials using the single-component presentation set in contact with ambient liquids are less antimicrobial than materials mixed with water [13]. AHBC is a ready-to-use sealer, and its setting reaction begins as soon as it obtains enough humidity; therefore, its hydration could have been delayed with respect to BR [11,16]. It also contained a lower percentage of SC in its composition (5–15%) and demonstrated a lower release of Ca^2+^ over time [19]. All these factors may contribute to a poor effect in the short term. The long-term effects of AHBC could be attributed to its composition. This includes dimethyl sulfoxide (DMSO) (10–30%), an organic solvent that has shown analgesic, anti-inflammatory, and antimicrobial properties [27]. Even at low concentrations, DMSO is not inert, and in some contexts, it shows antibacterial properties, generating changes in cellular processes [28], so its presence in the environment where the sealer is located could affect bacterial viability. Accordingly, the release of DMSO over time might contribute to its antibacterial efficacy. The fact that AHBC includes zirconium dioxide (ZrO_2_) (50–75%) as a radiopacifier [19] also deserves mention. ZrO_2_ nanoparticles have demonstrated antibacterial properties against oral [29] and other bacteria [30] by attracting the negatively charged cell wall against positively charged Zr ions. It has been reported recently that the AHBC sealer is highly soluble [16,18,19,31], which is attributed to the lower percentage of tricalcium silicate cement present in the sealer. This could strongly promote the release of DMSO and Zr from the sealer.

Still, the results cannot necessarily be extrapolated to the clinical situation, which is acknowledged as a limitation. Future studies, including ex vivo and in vivo research, should continue evaluating the antimicrobial and cytotoxic properties of bioceramics over time, as in vitro conditions do not fully represent the complexity or variability of a clinical situation.

## 5. Conclusions

Under the experimental conditions of this study, in the short term, AH Plus Jet and BioRoot RCS showed antimicrobial efficacy. BioRoot RCS and AH Plus BC obtained the best antibiofilm activity over time.

## Figures and Tables

**Table 1:** Endodontic sealers.

Material (Lot Number)	Composition	Manipulation
AH Plus Jet (AH) (Dentsply Sirona, Ballaigues, Switzerland) (2304000347)	Paste A: bisphenol-A epoxy resin (25–50%), epoxy resin bisphenol-F (2.5–10%), calcium tungstate, zirconium oxide, silica, iron oxide pigments Paste B: N,N′-dibenzyl-5-oxanonandiamine-1,9 (10–25%), amantadine (2.5–10%), calcium tungstate, zirconium oxide, silica, silicone oil	Dual self-mixing syringe
AH Plus Bioceramic (AHBC) (Dentsply Sirona, Ballaigues, Switzerland) (KI221118)	Zirconium dioxide (50–75%), tricalcium silicate (5–15%), dimethyl sulfoxide (10–30%), lithium carbonate (<0.5%), thickening agent (<6%)	Pre-mixed single syringe
Bioroot RCS (BR) (Septodont, Saint-Maur-des-Fossés, France) (B29755)	Powder: tricalcium silicate (25–50%), zirconium oxide (25–50%), povidone Liquid: water, calcium chloride, water-soluble polymer	Powder–liquid: 1 tablespoon of powder and 5 drops of liquid mixed for 60 s

**Table 2:** Antimicrobial activity of endodontic sealers against polymicrobial biofilms determined by the ATP test. Mean (DE) (n = 10/group/time).

Group	ATP Test Relative Light Units (RLUs)		
	1 Week	6 Weeks	Comparison *p*-Value **
AH Plus Jet	42,931.2 (11,093.5) ^a,1^	38,245.7 (11,429.3) ^a,2^	0.023
AH Plus BC	201,947.8 (49,986.7) ^b,d,1^	23,395.7 (7108.9) ^b,2^	<0.001
BioRoot RCS	142,614.2 (49,986.1) ^c,1^	20,216.5 (5455.3) ^b,2^	<0.001
Control	228,075.5 (71,556.6) ^d,1^	133,743.3 (21,459.7) ^c,2^	
Comparison *p*-value *	<0.001	<0.001	

* Global comparison determined via an ANOVA with Welch’s correction. Read vertically, the same letters in superscript show no significant differences determined by the Games–Howell test. ** A two-to-two comparison of 1- and 6-week RLUs. Read horizontally, the same numbers show no significant differences determined by the Student’s *t*-test.

**Table 3:** Antimicrobial activity of endodontic sealers against polymicrobial biofilms determined by flow cytometry. Mean (SD) n = 10/group/time.

Group	Cells with Damaged Membrane/mL		
	1 Week	6 Weeks	Comparison *p*-Value **
AH Plus Jet	12,115 (4939) ^a,1^	8557.6 (2950.3) ^a,d,1^	0.066
AH Plus BC	4540.4 (1955.9) ^b,d,1^	13,878.1 (3287.7) ^b,2^	<0.001
BioRoot RCS	15,544.1 (1793) ^c,1^	19,288.0 (903.3) ^c,2^	<0.001
Control	6281 (2582.6) ^d^	8266.5 (2416.1) ^d^	
Comparison *p*-value *	<0.001	<0.001	

* Global comparison determined by an ANOVA. Read vertically, the same letters in superscript show no significant differences determined by Duncan’s test. ** A two-to-two comparison of absolute values of cells with a damaged membrane at 1 and 6 weeks. Read horizontally, the same numbers show no significant differences determined by the Student’s *t*-test.

## Data Availability

The data presented in this study are available upon reasonable request from the corresponding author due to privacy.
